# Surveillance of Viral Respiratory Infections in the Neonatal Intensive Care Unit—Evolution in the Last 5 Years

**DOI:** 10.3390/pathogens12050644

**Published:** 2023-04-27

**Authors:** Blanca Bravo-Queipo-de-Llano, Laura Sánchez García, Inmaculada Casas, Francisco Pozo, Leticia La Banda, Sonia Alcolea, Jorge Atucha, Rocío Sánchez-León, Adelina Pellicer, Cristina Calvo

**Affiliations:** 1 Paediatric Infectious and Tropical Diseases Department, Hospital Universitario La Paz, Hospital La Paz Institute for Health Research (IdiPAZ Foundation), 28046 Madrid, Spain; 2 Department of Neonatology, Hospital Universitario La Paz, Hospital La Paz Institute for Health Research (IdiPAZ Foundation), 28046 Madrid, Spain; 3 Respiratory Viruses and Influenza Unit, National Centre of Microbiology, 28222 Madrid, Spain; 4 Centro de Investigación Biomédica en Red en Epidemiología y Salud Pública (CIBERESP), Instituto de Salud Carlos III, 28222 Madrid, Spain; 5 Centro de Investigación Biomédica en Red en Enfermedades Infecciosas (CIBERINFEC), Instituto de Salud Carlos III, 28222 Madrid, Spain; 6 Traslational Research Network in Pediatric Infectious Diseases (RITIP), Universidad Autónoma de Madrid, 28049 Madrid, Spain

**Keywords:** infectious diseases, respiratory viruses, COVID-19 pandemic, neonatal infections, neonatology

## Abstract

Viral respiratory infections (VRIs) in very low birthweight infants can be associated with high rates of morbidity. The COVID-19 pandemic has exerted a strong impact on viral circulation. The purpose of this study is to report on VRIs during NICU admission in infants below 32 weeks’ gestation and compare data collected between the pre-and post-COVID-19 pandemic periods. A prospective surveillance study was conducted at a tertiary NICU between April 2016 and June 2022. The COVID-19 post-pandemic period was established as being from March 2020 onwards. Respiratory virus detection was performed by real-time multiplex PCR assays in nasopharyngeal aspirates (NPAs). A total of 366 infants were enrolled. There were no statistical differences between periods regarding infants’ birth weight, gestational age, gender distribution, or rates of bronchopulmonary dysplasia. Among the 1589 NPA collected during the pre-COVID-19 period, 8.9% were positive, and among the 1147 NPA collected during the post-pandemic period, only 3% were positive (*p* < 0.005). The type of viruses detected did not differ according to the study period (pre-COVID19 vs. post-COVID-19): rhinovirus (49.5% vs. 37.5%), adenovirus (22.6% vs. 25%), and human coronavirus (12.9% vs. 16.7%). SARS-CoV-2 was only detected in one patient. In conclusion, the viral profile causing VRI during the pre-COVID-19 and post-COVID-19 era was similar. However, the total number of VRI dropped significantly, most probably due to the global increase in infection prevention measures.

## 1. Introduction

Viral respiratory infections (VRIs) are very common among children, often causing mild disease, although they can be asymptomatic. However, these infections are associated with higher rates of morbidity if they occur in preterm infants with a greater need for supplementary oxygen, later acquisition of full enteral nutrition, and longer hospital stay, especially in those who suffer bronchopulmonary dysplasia [1,2,3].

Despite the general thought that Respiratory Syncytial Virus (RSV) was the most frequent virus associated with classical respiratory features in infants, there is growing evidence to suggest that many other viruses are involved [4,5,6]. A recent study conducted by our group described the variety of viruses identified in a prospective observational cohort of very low birth weight (VLBW) infants of less than 32 weeks of gestation admitted to the neonatal intensive care unit (NICU) between April 2016 and March 2018, where the most frequently identified virus was rhinovirus (hRV) (58%), followed by adenovirus (AdV) (31%). No difference in clinical expression associated with the aetiological agent was found [1].

Since 2020, the COVID-19 pandemic and related mitigation strategies have exerted a strong impact on the circulation of influenza, RSV, and other respiratory viruses, with a sharp drop between mid-March 2020 and mid-April 2020 [7]. Many authors [8,9,10] have reported a similar decrease in virus detection in children around the world. Current data indicate a low risk of SARS-CoV-2 transmission from positive pregnant women to their offspring, and once they are born, if infected, the neonatal disease usually ranges from asymptomatic to mildly symptomatic [11,12]. However, information on the behaviour and detection of other respiratory viruses in neonates as a result of the COVID-19 pandemic is lacking. Despite their vulnerability to infection, the systematic research of viruses among VLBW infants during NICU admission is not clinical routine in most units, and viruses are frequently not thought about in cases of apneas, increased needs of supplementary oxygen or feeding difficulties. Clinical features of VRI, in fact, are non-specific and mirror bacterial infection [1]. The purpose of this study is to report on a systematic VRI surveillance in VLBW infants during NICU admission, with a focus on the eventual evolving pattern of viral-type infection considering the pre- and post-COVID-19 era.

## 2. Materials and Methods

### 2.1. Study Population

A prospective surveillance study was conducted at the Department of Neonatology at La Paz University Hospital (Madrid, Spain) between April 2016 and June 2022. Data on the viral respiratory infections between April 2016 and March 2018 have already been published by our group [1]. Our NICU covers 5000 births with approximately 100 admissions below 32 weeks of gestation per year. Those admitted to the NICU within the first 72 h from birth, for whom we had signed informed parental consent, were enrolled in the study. Exclusion criteria were admission beyond 72 h of life, death within the first week, severe congenital malformations, or declined parental consent. Each infant’s information was treated anonymously.

### 2.2. Study Procedures

Nasopharyngeal aspirates (NPAs) for respiratory viruses were collected within the first 72 h after birth and then weekly until discharge. Additional NPAs were collected in the case of respiratory symptoms or with the non-specific worsening of clinical condition leading to an initial sepsis workup. Respiratory features included cough, increased respiratory workload, tachypnoea, nasopharyngeal secretions, fever, desaturations, or increased oxygen demands, bradycardia, or apnoea. A new episode was considered in case of positive NPA after at least two previous negative samples and a minimum of 21 days after the last positive one or when positive NPA to a different virus species was detected. Clinical data were extracted from clinical records and prospectively registered. Epidemiological survey was undertaken in positive NPAs to rule out nosocomial outbreak (rooming-in with other positive cases).

### 2.3. Microbiological Assay

NPAs were analysed at the Influenza and Respiratory Viruses Laboratory at the National Centre for Microbiology (Madrid, Spain). Samples were processed within 24 h after collection. Upon reception, three aliquots were prepared and stored at −80 °C. Both the reception and the NPA sample processing areas were separated from those defined as working areas. RNA and DNA from 200 μL aliquots of NPA were extracted using the QIAamp MinElute Virus Spin Kit in an automated extractor (QIAcube, Qiagen, Valencia, Spain). Respiratory virus detection was performed by four independent real-time multiplex PCR (RT-PCR) assays using the SuperScript III Platinum One-Step Quantitative RT-PCR System (Invitrogen^®^, Waltham, MA, USA). The first assay detected Influenza A, B, and C viruses; the second assay detected parainfluenza viruses 1 to 4 (PIV), hRV and enteroviruses; the third assay detected RSV types A and B, human metapneumovirus (hMPV), human bocavirus (hBoV), and AdV. Human coronavirus (HCoV) was investigated using a generic RT-PCR that was able to detect human alpha and beta coronavirus, HCoV 229E/HCoV NL63, and HCoV OC43/HCoV HKU1, respectively. The primers and Taqman probes used in the study had already been reported by the study investigators [13]. In addition, detection of SARS-CoV-2 was performed on an extracted RNA from NPAs from 2020 using a real-time RT-PCR assay based on the method designed by Corman et al. [14] for the specific amplification of the E gene using the One-Step RT-PCR Kit (NZYTech, Lisbon, Portugal). This method was adapted to our laboratory, including the amplification of an internal control from the sample in a multiplex way. Assay sensitivity was regularly assessed to check for potential failures in specificity associated with viral variability. Quality controls organized by ECDC/WHO and QCMD were received annually to check the sensitivity and specificity of all of the tests used.

### 2.4. Data Analysis

Information was analysed using the IBM SPSS Statistics version 26 software (Madrid, Spain). Continuous variables were presented as means and standard deviations (SD), or medians and interquartile ranges (IQR). Categorical variables were expressed as frequencies and percentages.

Normal distribution of groups was analysed via the Kolmogorov–Smirnov test and homoscedasticity via Levene’s test. Associations among independent continuous variables between the pre-COVID-19 period (April 2016 to February 2020) and post-COVID-19 period (March 2020 to June 2022) were compared using Student’s *t*-test (or Mann–Whitney test when appropriate as a non-parametrical test). Categorical variables were statistically analysed by the Chi-squared test (or Fisher’s exact test when the sample size was small). Statistical significance was defined as a level of *p*-value < 0.05.

## 3. Results

During the study period, 366 infants below 32 weeks of gestation were eligible for the study: 238 infants started follow-up during the pre-COVID-19 period and 128 during the post-COVID-19 pandemic period. Four patients died in the first period and three in the second period. There were no patients lost to follow-up.

Weight at birth showed a normal distribution in groups pre-COVID-19 and post-COVID-19 (Kolmogorov–Smirnov normality test, *p* = 0.2 in both periods; equality of variances Levene’s test, *p* = 0.429) while gestational age did not show a normal distribution (Kolmogorov–Smirnov normality test, *p* < 0.002) in both periods, pre- and post-COVID-19.

Mean birth weight (1119.8 ± 327.7 grams vs. 1136.8 ± 346.3), gestational age (29 weeks, IQR 26.9–30.3 vs. 29, IQR 27–30.6), gender distribution (males 50% vs. 57%), and bronchopulmonary dysplasia (BPD) rates (48.5% vs. 36.6%) did not statistically differ between the pre-COVID-19 and post-COVID-19 pandemic cohorts (*p* > 0.05).

During the pre-COVID-19 period, 1589 NPAs were collected, yielding 8.9% positive results. The median number of samples per patient was six (IQR 4.5–9). Ninety-three infants (39% of the pre-COVID-19 cohort) had at least a positive sample. The most frequently isolated species were hRV (49.5%), followed by AdV (22.6%), and HCoV (12.9%). Positive NPA occurred in clusters during the autumn and winter seasons.

During the post- COVID-19 period, 1147 NPA were collected, with 3% positive results, representing a statistically significant decrease with respect to the pre-pandemic period (Chi-squared test, *p* < 0.005). The median number of samples per patient was eight (IQR 6–11). Twenty-four infants (18.8% of the post-COVID-19 cohort) had at least a positive sample. The profile of isolated species was very similar to that of the pre-pandemic period, with no statistically significant differences between periods regarding hRV (37.5%), AdV (25%), and HCoV (16.7%)—the most prevalent viruses (*p* > 0.05).

**Figure 1:**
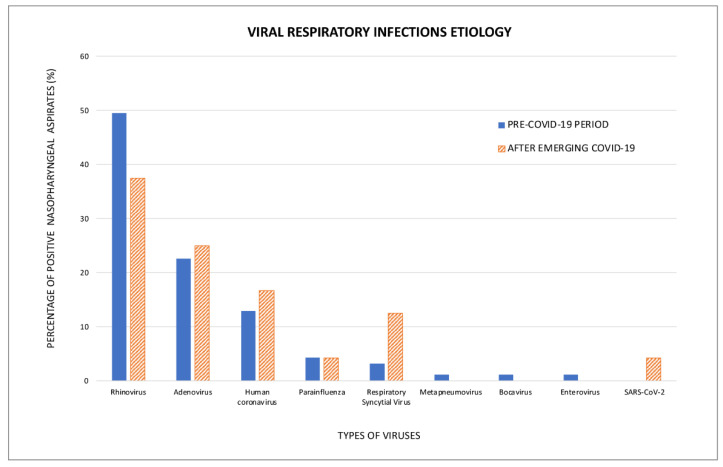
The aetiology of viral respiratory infections.

**Figure 2:**
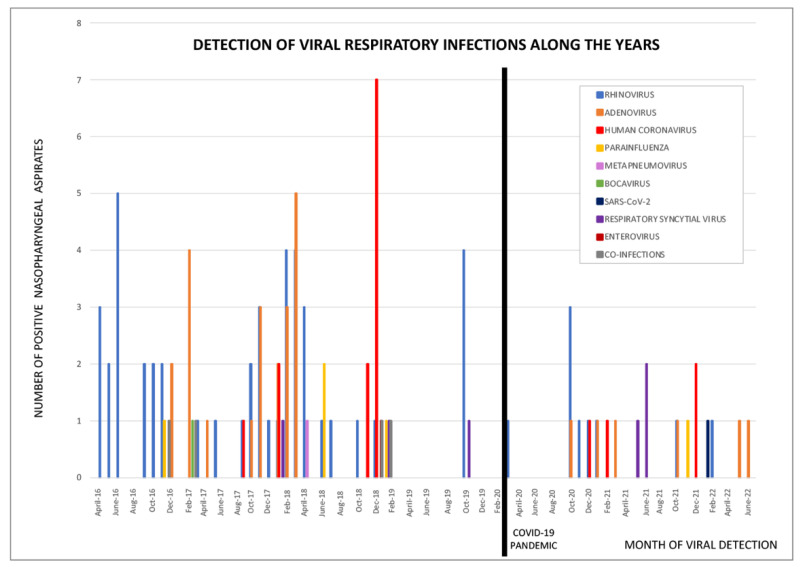
Detection of viral respiratory infections along the years.

PIV and RSV were studied together due to the small number of positive results irrespective of the study period (Fisher’s exact test, *p* = 0.233); however, RSV was found to have a peak in June 2021, which coincided with a summer outbreak in Spain. SARS-CoV-2 was only detected in one patient (4.2%). The variety of viruses identified is displayed in Figure 1. The prevalence of other viral species was scarce and only present during the pre-COVID-19 pandemic, therefore preventing group comparisons between study periods. The distribution of viral species across the study period is displayed in Figure 2.

## 4. Discussion

This study reports on the viral cause of respiratory infections in preterm infants admitted to the NICU and compares data collected between the pre- and post-COVID-19 pandemic periods. The systematic surveillance of VRI in VLBW infants disclosed a significant decrease in overall VRI within the NICU during the post-pandemic period.

The transmission of respiratory viruses occurs either via aerosol route or direct contact, with the transfer of viruses usually occurring due to contaminated surfaces being touched by the hands during routine activities. Contamination through parents and staff has been described in hospitalized neonates [15]. Since the emergence of COVID-19, we have observed a significant reduction in VRI. During the year 2020, the overall number of newborns with lower respiratory tract infection (LRTI) declined significantly, and by the end of the year, some sporadic non-RSV cases were detected, particularly hRV and hMPV infections [16]. Of note, the observed decrease in VRI rates coincides with the enhancement of infection prevention measures all around the world, including physical distancing, hygiene promotion, and targeted restrictions on gathering and movement [17].

In our series, we have found a similar profile of types of viruses causing VRI throughout the observed years independent of the COVID-19 pandemic. The most frequently isolated species were hRV, followed by AdV and HCoV. In spite of the general thought that RSV is the virus most frequently associated with classical respiratory features in premature infants, studies such as ours, in which specific surveillance has been performed, have shown a higher prevalence of non-RSV-related aetiology. These findings are in alignment with other reports wherein nosocomial VRIs in neonates were mostly associated with HCoV infection [15] or hRV, hMPV, PIV, influenza, hBoV, and HCoV [16]. These VRIs can cause severe respiratory disease, and in the preterm infant, hRV may be a determinant in the development of complicated LRTIs (apnoea, atelectasis, bacterial superinfection, and sepsis).

Although nosocomial VRIs in the NICU are usually considered anecdotal, a recent study from our group found that 38% of infants born below 32 weeks of gestation have at least one positive NPA sample during their NICU admission, and a significant number of them are free of clinical signs or associate non-specific clinical signs that could be interpreted as prematurity-related events or bacterial infection rather than LRTI caused by viral pathogens [1]. A well stablished surveillance routine, using advanced PCR diagnostic technology, will contribute to an earlier and more accurate detection of respiratory viruses compared to traditional antigen tests and virus cultures [18]. VRIs are an important determinant of morbidity in vulnerable populations [2,19] and represent an immunologic challenge [20]. Our research suggests that premature infants with symptomatic VRIs during NICU admission have an increased risk of developing recurrent wheezing during the first two years of age. There is an increasing amount evidence to suggest that early life VRIs favour the development of an abnormal respiratory epithelial layer, leading to deviations in T cell maturation along the pro-allergic T2 pathway [21].

The main limitation of our study is the relatively small sample size of positive NPA obtained, making group comparisons difficult on some occasions. However, the strengths rely on a systematic prospective assessment of a large and uniform cohort of preterm infants during NICU admission.

In conclusion, we did not find much statistically significant difference in virus circulation since the COVID-19 pandemic. However, the total number of VRIs has significantly dropped during the post-pandemic period, most probably due to the global increase in infection prevention measures.

## Data Availability

The data presented in this study are available on request from the corresponding author.
